# Inhibition of NF-κB signaling in *IKKβ^F/F^;LysM Cre* mice causes motor deficits but does not alter pathogenesis of Spinocerebellar ataxia type 1

**DOI:** 10.1371/journal.pone.0200013

**Published:** 2018-07-05

**Authors:** Austin Ferro, Wenhui Qu, Abigail Lukowicz, Daniel Svedberg, Andrea Johnson, Marija Cvetanovic

**Affiliations:** 1 Department of Neuroscience, University of Minnesota, Minneapolis, Minnesota, United States of America; 2 Institute for Translational Neuroscience, University of Minnesota, Minneapolis, Minnesota, United States of America; Stony Brook University, UNITED STATES

## Abstract

Spinocerebellar Ataxia type 1 (SCA1) is a fatal neurodegenerative genetic disease that is characterized by pronounced neuronal loss and gliosis in the cerebellum. We have previously demonstrated microglial activation, measured as an increase in microglial density in cerebellar cortex and an increase in the production of pro-inflammatory cytokines, including tumor necrosis factor alpha (TNF-α), in the cerebellum of the *ATXN1[82Q]* transgenic mouse model of SCA1. To examine the role of activated state of microglia in SCA1, we used a Cre-Lox approach with *IKKβ*^*F/F*^*;LysM Cre* mice intended to reduce inflammatory NF-κB signaling, selectively in microglia. *ATXN1[82Q];IKKβ*^*F/F*^*;LysM Cre* mice showed reduced cerebellar microglial density and production of TNFα compared to *ATXN1[82Q]* mice, yet reducing NF-κB did not ameliorate motor impairments and cerebellar cellular pathologies. Unexpectedly, at 12 weeks of age, control *IKKβ*^*F/F*^*;LysM Cre* mice showed motor deficits equal to *ATXN1[82Q]* mice that were dissociated from any obvious neurodegenerative changes in the cerebellum, but were rather associated with a developmental impairment that presented as a retention of climbing fiber synaptic terminals on the soma of Purkinje neurons. These results indicate that NF-κB signaling is required for increase in microglial numbers and TNF-α production in the cerebella of *ATXN1[82Q]* mouse model of SCA1. Furthermore, these results elucidate a novel role of canonical NF-κB signaling in pruning of surplus synapses on Purkinje neurons in the cerebellum during development.

## Introduction

Spinocerebellar ataxia type 1 (SCA1) is an autosomal dominant neurodegenerative disease caused by the abnormal expansion of CAG repeats in the coding region of *Ataxin1 (ATXN1)* gene [1]. The CAG repeat is translated into an expanded polyglutamine (polyQ) track in the ATXN1 protein [2], which places SCA1 into the family of polyglutamine diseases that also includes SCA 2, 3, 6, 7, 17, Kennedy disease, Huntington’s disease, and dentatorubropallidoluysian atrophy [3]. More than 39 uninterrupted CAG repeats in the *ATXN1* gene causes SCA1, with the initial symptoms of ataxia, defined by movement and balance deficits, normally presenting in the patient’s mid-thirties. Motor deficits progressively worsen until premature death, commonly due to pulmonary compromise, 10–20 years from the disease onset [4]. There is no disease modifying therapy or cure for SCA1.

*In vivo* magnetic resonance imaging (MRI) demonstrated severe atrophy of the cerebellum and brain stem [5], and proton magnetic resonance spectroscopy (1H MRS) revealed neurochemical alterations in the cerebellum indicative of neuronal dysfunction/neurodegeneration and gliosis both in SCA1 patients and mouse models [6][7]. Postmortem analysis supports a predominantly cerebellar pathology with severe loss of Purkinje neurons and gliosis in the cerebellar cortex [8]. These pathological changes in the cerebellum are the likely cause of motor deficits observed in patients. In addition, longitudinal imaging studies demonstrated that cerebellar gliosis and neuronal dysfunction/degeneration correlate well with the clinical progression of disease [9][10][6][7]. We previously demonstrated that cerebellar microglia show signs of activation, defined by an increase in microglial density and hypertrophy of the soma and processes [11], in the *ATXN1[82Q]* transgenic mouse model of the SCA1 [10]. Furthermore, we have found that microglial activation was associated with increase in the production of pro-inflammatory cytokines, including tumor necrosis factor alpha (TNFα), and was detectable prior to the loss of Purkinje neurons and motor deficits [10].

Microglia are resident phagocytic and immune cells of the central nervous system (CNS) that constitute about 5–12% of the brain glia [12]. Microglia are derived from the primitive macrophages in the yolk sac that colonize the neuroepithelium at E9.5 [13]. Microglia have significant roles in neuronal development [14][15][16][17][18], control of homeostasis of the healthy adult brain[19] and have been shown to have profound functions in most neurodegenerative diseases [20][21][22]. During development microglia play an important role in inducing and engulfing apoptotic neurons and glia as well as pruning immature surplus synapses to shape neuronal connectivity. However, while recent study demonstrated presence of phagocytic cups in microglia of developing rat cerebellum [18], little is known about their role in cerebellar development.

Upon sensing a change in brain homeostasis during injury or disease, microglia undergo morphological and functional change termed activation. While microglial activation is diverse and depends on the context of the insult and brain region, it often includes increase in microglial density, enlargement of cell bodies, and release of inflammatory cytokines that can modulate functions of astrocytes and neurons, such as brain-derived neurotrophic factor (BDNF) and TNFα [23][24][25][26][27][28][29][30][31]. While microglial activation has been described in many neurodegenerative disease, its functional role remains unclear as both beneficial and harmful effects have been reported [32][33][22][34].

Putative beneficial functions of reactive microglia can be accomplished through secretion neurotrophic factors such as BDNF and through the active removal of dying cells, non-functional synapses and extracellular aggregates via phagocytosis [22]. Activated microglia can be harmful by inappropriately removing functional synapses and thereby damaging the neuronal networks, and by inducing potentially neurotoxic inflammation. The microglia-induced neuroinflammation is largely mediated by the release of cytokines, including TNFα that have been shown to worsen outcome in various neurological disorders, including Alzheimer’s disease (AD), Multiple Sclerosis (MS), and Parkinson’s disease (PD) [35][36][37][38]. Microglial inflammation is modulated by the activity of the transcriptional factor nuclear factor kappa (NF-κB), a master regulator of inflammation and a well-known regulator of TNFα expression [39]. Under normal physiological conditions, most of NF-κB is kept in the cytoplasm by being bound to the inhibitor of κB (IκB) proteins, therefore limiting NF-κB’s transcriptional activity [40]. Inflammatory stimuli commonly activate NF-κB through the classical inhibitor of nuclear factor kappa-B kinase subunit beta (IKKβ) pathway, in which inducing stimuli trigger phosphorylation of catalytic unit of IκB kinase (IKK) complex, IKKβ subunit at Ser 180 [41][42]. Activated IKKβ in turn phosphorylates IκB proteins leading to their ubiquitination and degradation [39], thus releasing NF-κB dimers to translocate to the nucleus and promote transcription of target genes. Compared to other brain regions, under physiological conditions cerebellar microglia exhibit a more alert immune phenotype that is in part regulated by NF-κB proteins [43]. However, how the NF-κB pathway in cerebellar microglia contributes to the pathogenesis of SCA1 is unknown.

Here, we used Cre-Lox approach to inhibit NF-κB signaling in microglia of SCA1 mice. We crossed *IKKβ*^*F/F*^ mice [44] with *LysM Cre* mice [45][44][46] to cause microglia selective deletion of IKKβ and transgenic mouse model of SCA1, *ATXN1[82Q]* mice that express mutant ATXN1 with 82 glutamine repeats only in Purkinje neurons (**S1 Fig**) [47]. We have found that *ATXN1[82Q];IKKβ*^*F/F*^;*LysM Cre* mice had decreased density of microglia and expression of TNFα compared to *ATXN1[82Q]* littermates, suggesting that NF-κB pathway regulates early microglial recruitment and TNFα production in cerebella of SCA1 mice. However, onset and severity of SCA1 pathogenesis were not significantly altered in *ATXN1[82Q];IKKβ*^*F/F*^;*LysM Cre* mice suggesting that early microglial neuroinflammation may not contribute to the pathogenesis of SCA1. This interpretation was complicated as we have also found that control *IKKβ*^*F/F*^;*LysM Cre* mice exhibited motor deficits at 3 months of age associated with a reduction in developmental pruning of surplus synapses on soma of Purkinje neurons. Moreover, using reporter mice to examine Cre expression, we have found that despite *LysM Cre* line being previously used as a microglia specific in the CNS, Cre activity was also evident in cerebellar neurons, including Purkinje neurons.

## Results

### NF-κB pathway contributes to increase in microglial density and TNF-α production in SCA1 mice

We used a mouse genetic approach to reduce activation of the pro-inflammatory transcriptional regulator NF-κB selectively in microglia of SCA1 mice. The classical pathway of NF-κB signaling involves activation of IκB kinase (IKKβ) that phosphorylates IκB proteins marking them for ubiquitination and degradation. This frees NF-κB to translocate into the nucleus and mediate gene expression. We deleted the catalytic subunit of *IKKβ* using a Cre-LoxP approach and produced the *IKKβ*^*F/F*^*;LysM Cre* mouse line, in which the putative microglia and macrophage specific *Lysozyme M* promoter drives the expression of Cre-recombinase [45][48] to delete the exon 3 of *IKKβ* [44][49](**S1 Fig**). These mice were crossed with Purkinje neuron specific transgenic mouse model of SCA1, *ATXN1[82Q]* mice. *ATXN1[82Q]* mice demonstrate many features of SCA1 including increase in the cerebellar microglial density at four weeks of age and motor deficits and cerebellar pathology at 12 weeks [10].

**Fig 1:**
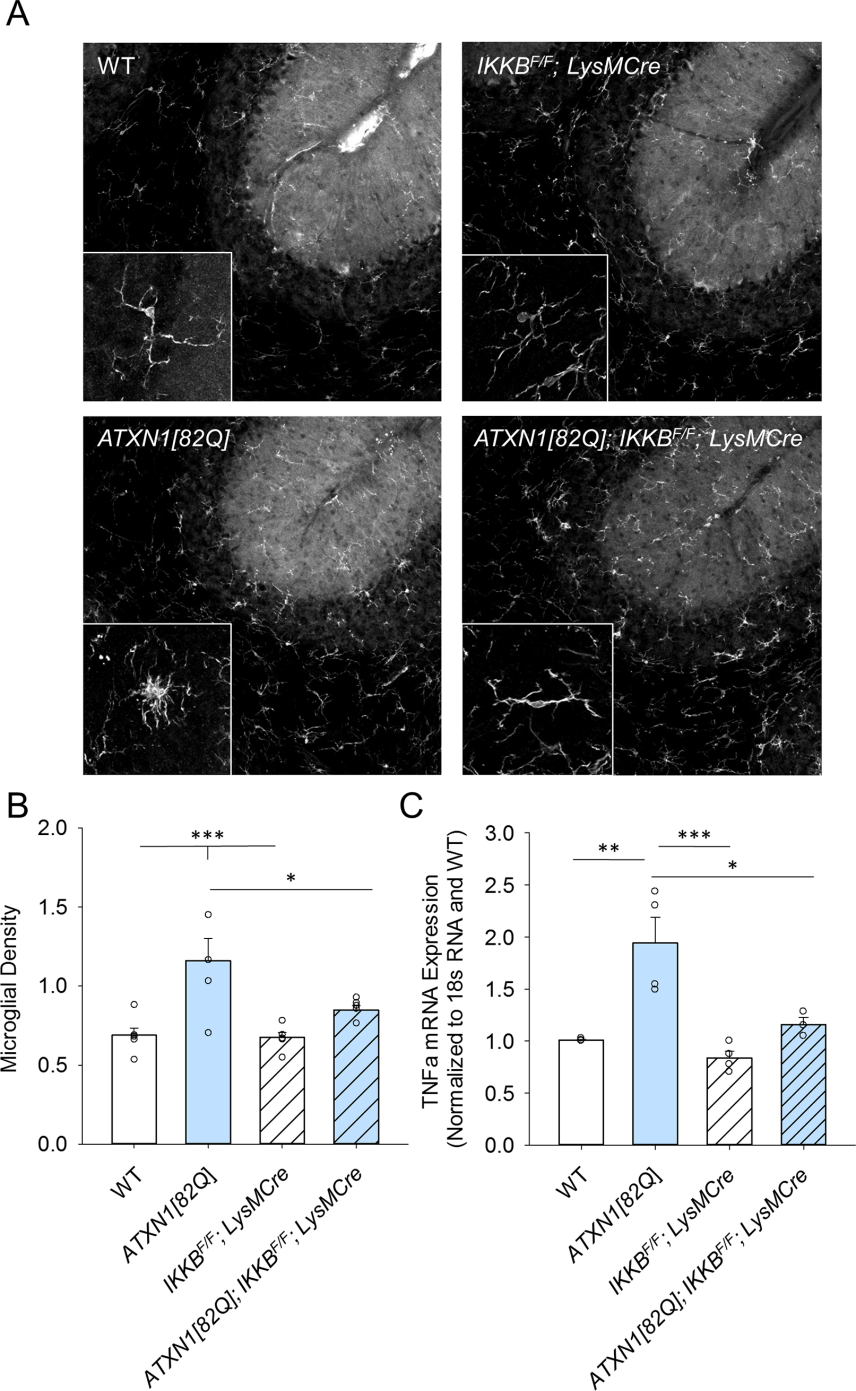
Microglial density and TNFα production in *ATXN1[82Q]; IKKβ*^*F/F*^*;LysM Cre* mice. **A.** Cerebella of 3-month–old mice were used for IHC with Iba1-antibody. Representative image of microglia specific Iba-1 staining. **Insets of** higher magnification images illustrating microglial morphology. **B.** Quantification of microglial density in the molecular layer (N ≥ 4 per each genotype), one-way ANOVA followed by Tukey’s HSD post-hoc test. * P < 0.05 **C.** RNA was extracted from the cerebella of 3-month–old mice and RT-qPCR was used to determine expression of TNFα (with reference to control treated wild type and normalized to 18S RNA). Each dot represents one mouse, and values indicate mean ± SEM, one-way ANOVA followed by Bonfferoni post-hoc test. * P < 0.05, ** P < 0.01, *** P < 0.0001. Open bars are WT controls, light blue bars are *ATXN1[82Q]* mice, and hashed bars indicate the presence of *IKKβ*^*F/F*^*;LysM Cre*.

We examined the effect of NF-κB inhibition on microglial density and morphology by performing immunohistochemistry for microglial marker ionic calcium binding protein (Iba1) (**Fig 1A**). As Cre recombinase is expressed throughout the brain in *LysM Cre* line it could potentially affect microglia in other brain regions involved in movement control, such as striatum. Thus, we first quantified microglia in the striatum of control and *IKKβ*^*F/F*^*;LysM Cre* mice to control for these extra-cerebellar changes. We found that microglial density and morphology were indistinguishable in the striatum of *IKKβ*^*F/F*^*;LysM Cre* mice compared to wild-type littermate controls (**S2 Fig**), indicating that reduced NF-κB signaling does not alter microglial density in the striatum. Yet, we found significant reduction in the number of Iba1 positive microglia in the cerebella of *ATXN1[82Q];IKKβ*^*F/F*^*;LysM Cre* mice compared to *ATXN1[82Q]* control mice (**Fig 1B,** one-way ANOVA with Bonfferoni t-test, p = 0.031). In addition, microglia in the cerebella of *ATXN1[82Q];IKKβ*^*F/F*^*;LysM Cre* mice did not seem to exhibit enlarged morphology seen in *ATXN1[82Q]* mice microglia (**Fig 1A insets**). These results suggest that the canonical NF-κB pathway is involved in regulating microglial density and morphology in SCA1.

We have previously demonstrated that microglia produce the majority of pro-inflammatory cytokine TNFα in SCA1 mice. Thereby we examined whether expression of TNFα was altered in the cerebella of *ATXN1[82Q];IKKβ*^*F/F*^*;LysM Cre* mice [50]. Using quantitative reverse transcriptase polymerase chain reaction (RT-qPCR), we detected a decrease in the expression of TNFα in the cerebella of *ATXN1[82Q]; IKKβ*^*F/F*^*;LysM Cre* mice compared to *ATXN1[82Q]* mice suggesting that NF-κB pathway regulates the increase in TNFα (**Fig 1C**, *ATXN1[82Q]*: 1.987, N = 4, *ATXN1[82Q]; IKKβ*^*F/F*^*;LysM Cre*: 1.46, N = 3, one-way ANOVA with Bonfferoni t-test, p = 0.019).

### Inhibition of NF-κB is sufficient to cause motor deficits in *IKKβ*^*F/F*^*;LysM Cre* mice but does not alter SCA1 motor abnormalities

**Fig 2:**
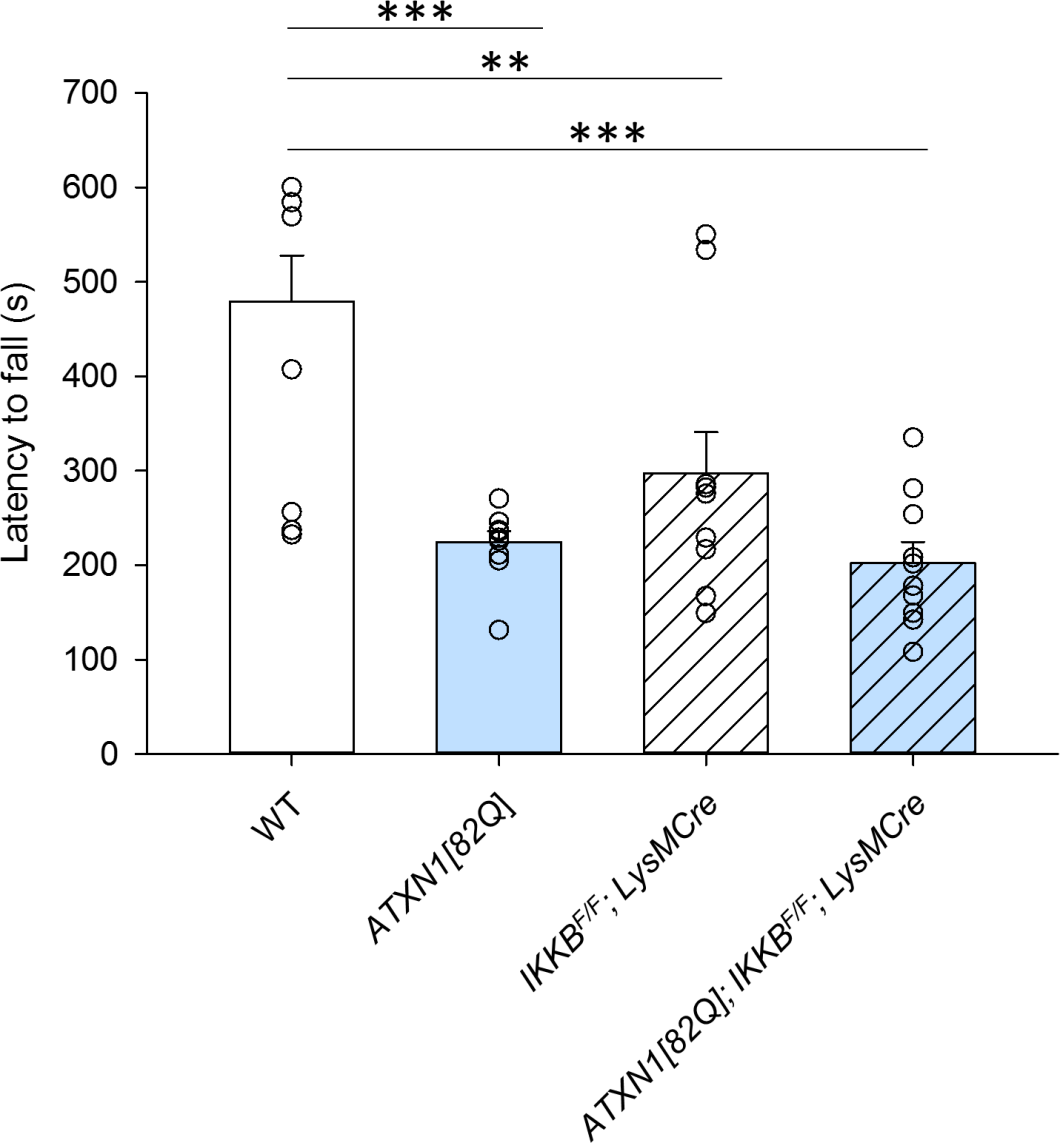
Motor performance of *IKKβ*^*F/F*^*;LysM Cre* and *ATXN1[82Q]; IKKβ*^*F/F*^*;LysM Cre* mice. Mice were tested on a rotarod at three months of age. Each dot represents one mouse, and values indicate mean ± SEM, one-way ANOVA followed by Bonfferoni post-hoc test. * P < 0.05, ** P < 0.01, *** P < 0.0001. Open bars are WT controls, light blue bars are *ATXN1[82Q]* mice, and hashed bars indicate the presence of *IKKβ*^*F/F*^*;LysM Cre*.

We next determined whether reducing microglial NF-κB signaling affects motor deficits characteristic for SCA1. One of the earliest symptoms of SCA1 is ataxia, which refers to a loss of motor control and balance. Ataxia in mice can be quantified by the performance on the accelerating rotating rod (rotarod) test [51], where latency to fall of the rotarod is measured. Mice with cerebellar deficits, such as SCA1 mice, classically have reduced latency to fall due to impaired balance [50]. At three months of age, the earliest age at which we can detect motor deficits in SCA1 mice, we found that *ATXN1[82Q];IKKβ*^*F/F*^*;LysM Cre* mice did not perform differently when compared to *ATXN1[82Q]* mice (**Fig 2**, *ATXN1[82Q]* mice 199.1s, N = 11, *ATXN1[82Q];IKKβ*^*F/F*^*;LysM Cre* 152.6s, N = 10, p > 0.05, one-way ANOVA with Bonferroni post-hoc testing).

Surprisingly, *IKKβ*^*F/F*^*;LysM Cre* mice were significantly impaired in rotarod performance when compared to control mice (**Fig 2**, WT mice 370s N = 11, *IKKβ*^*F/F*^*;LysM Cre* 198s, N = 10, p = 0.005, one-way ANOVA with Bonferroni post-hoc test). Moreover, haploinsufficient *IKKβ*^*F/WT*^*;LysM Cre* mice that carry only a single floxed IKKβ allele also demonstrated motor deficits (**S3 Fig**). These results suggest that reducing canonical NF-κB signaling in *IKKβ*^*F/F*^*;LysM Cre* mice is sufficient to cause motor impairments, but does not significantly affect SCA1-induced motor deficits.

**Fig 3:**
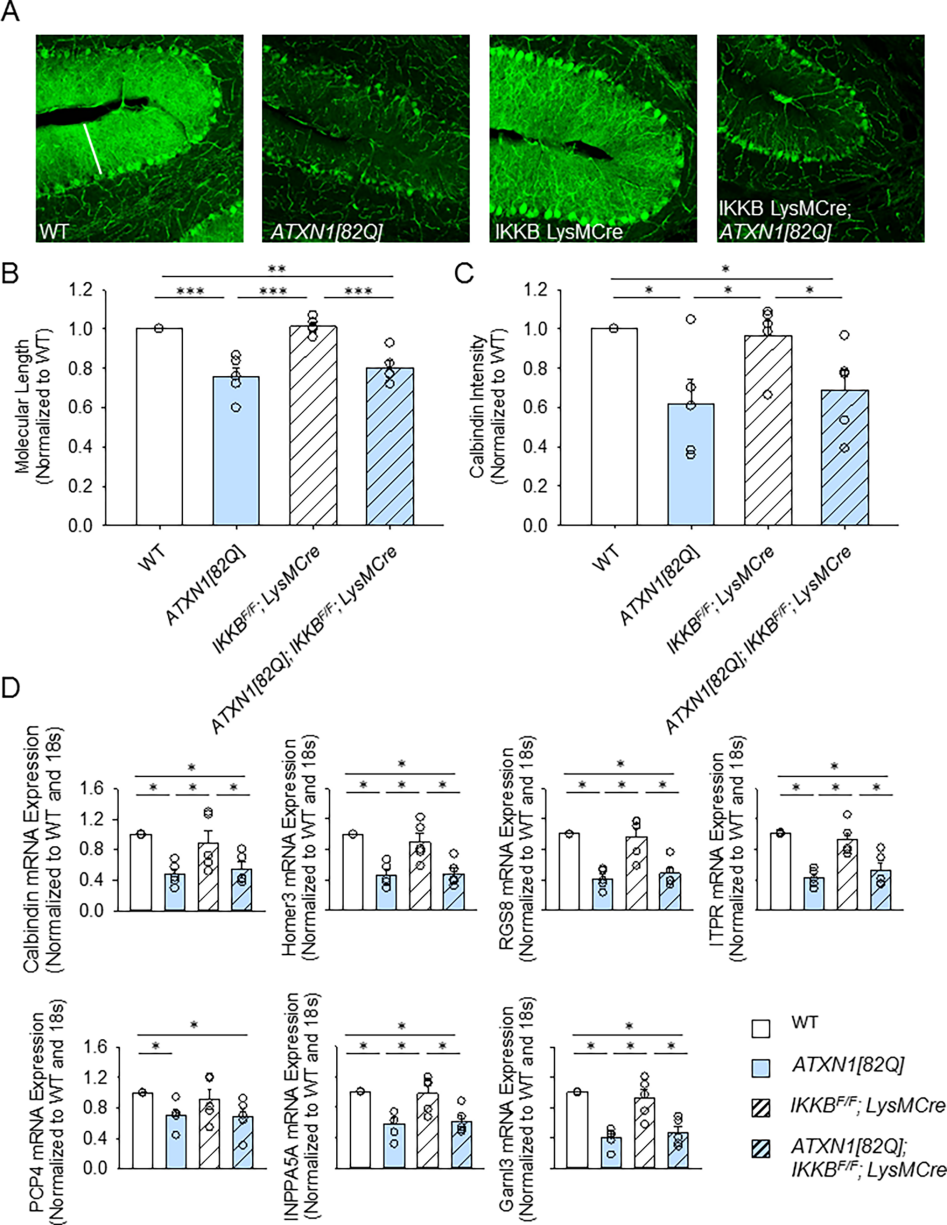
Cerebellar pathology in *IKKβ*^*F/F*^*;LysM Cre* and *ATXN1[82Q]; IKKβ*^*F/F*^*;LysM Cre* mice. **A.** Cerebella of 3-month-old mice stained with calbindin antibody, specific for Purkinje neurons. **B.** Quantification of the width of the molecular layer,* P < 0.05, Kruskal-Wallis test with Dunn’s test, N ≥ 4 per each genotype. **C.** Quantification of Calbindin intensity, * P < 0.05, Kruskal-Wallis test with Dunn’s test, N ≥ 4 per each genotype. **D.** RNA was extracted from the cerebella of 3-month–old mice and RT-qPCR was used to determine expression of Purkinje neuron genes belonging to Magenta cluster (with reference to control treated wild type littermates and normalized to 18S RNA). N ≥ 3 per each genotype. Each dot represents one mouse, and values indicate mean ± SEM. * P < 0.05, ** P < 0.01, *** P < 0.0001. Open bars are WT controls, light blue bars are *ATXN1[82Q]* mice, and hashed bars indicate the presence of *IKKβ*^*F/F*^*;LysM Cre*.

We next determined whether motor deficits in *IKKβ*^*F/F*^*;LysM Cre* are underlined by neurodegenerative changes in Purkinje neurons, such as atrophy of soma and dendrites previously described in SCA1 mice [52][53]. We used immunofluorescence of parasagittal cerebellar slices with antibody against calbindin, a marker of Purkinje neurons that labels their cell bodies and processes (**Fig 3A**). In SCA1 mice Purkinje neuron atrophy can be quantified as a decrease in the calbindin intensity and a decrease in the width of the molecular layer [54]. Neither the molecular layer width nor the intensity of calbindin staining were altered in *IKKβ*^*F/F*^*;LysM Cre* mice when compared to control WT mice (**Figs 3B and 3C**). We found a reduction in the width of the molecular layer in *ATXN1[82Q]* mice compared to WT mice; yet this decrease was not significantly altered in *ATXN1[82Q];IKKβ*^*F/F*^*;LysM Cre* mice (**Fig 3B**, normalized to N = 5 WT littermate controls *IKKβ*^*F/F*^*; LysM Cre* mice 0.96 ± 0.07, N = 5, *ATXN1[82Q]* mice 0.706 ± 0.12, N = 5, *ATXN1[82Q]; IKKβ*^*F/F*^*; LysM Cre* mice 0.68 ± 0.1, N = 5). Similar results were obtained for the intensity of calbindin staining that was decreased in both *ATXN1[82Q]* and *ATXN1[82Q]; IKKβ*^*F/F*^*;LysM Cre* mice (**Fig 3C**, normalized to N = 5 WT littermate controls *IKKβ*^*F/F*^*;LysM Cre* mice 0.96 ± 0.07, N = 5, *ATXN1[82Q]* mice 0.706 ±0.12, N = 5, *ATXN1[82Q]; IKKβ*^*F/F*^*;LysM Cre* mice 0.68 ± 0.1, N = 5).

A recent study identified a set of genes that have reduced expression in Purkinje neurons in SCA1 mice that correlates well with disease progression [51]. Therefore, we examined the expression of these markers of Purkinje cell degeneration in cerebellar extracts from each of our experimental mouse lines using qRT-PCR. We have found a decrease in the expression of these genes in *ATXN1[82Q]* mice, as would be expected based on the published data (**Fig 3D**) [51]. However, expression of these genes was indistinguishable in *ATXN1[82Q];IKKβ*^*F/F*^*;LysM Cre* and *ATXN1[82Q]* mice. Importantly, *IKKβ*^*F/F*^*; LysM Cre* mice also showed a slight but not significant decrease in gene expression compared to control wild-type littermates (**Fig 3D**, for example expression of *Homer3* normalized to N = 5 WT littermate controls in *IKKβ*^*F/F*^*;LysM Cre* mice 0.896 ± 0.01, N = 5, *ATXN1[82Q]* mice 0.465 ± 0.06, N = 5, *ATXN1[82Q]; IKKβ*^*F/F*^*;LysM Cre* mice 0.485 ± 0.07, N = 5, and for *Regulator of G protein signaling* (*Rgs8*) normalized to N = 5 WT littermate controls *IKKβ*^*F/F*^*; LysM Cre* mice 0.96 ± 0.11, N = 5, *ATXN1[82Q]* mice 0.41 ± 0.05, N = 5, *ATXN1[82Q]; IKKβ*^*F/F*^*;LysM Cre* mice 0.49 ± 0.07, N = 5).

The expression of ataxin-1 or the marker of post-synaptic area post-synaptic density 95 (PSD95) proteins was also not changed in *ATXN1[82Q];IKKβ*^*F/F*^*;LysM Cre* mice compared to *ATXN1[82Q]* mice (**S4 Fig**)[50]. Thus, our results indicate that reducing microglial NF-κB activity in *ATXN1[82Q];IKKβ*^*F/F*^*;LysM Cre* mice is not sufficient to alter behavioral and neurological pathology in SCA1 mice, and that motor deficits in *IKKβ*^*F/F*^*;LysM Cre* mice do not seem to be associated with degenerative changes in Purkinje neurons.

### Cerebellar astrogliosis is not altered in *ATXN1[82Q];IKKβ*^*F/F*^*;LysM Cre* mice

Astrocytes are another type of glial cells that are activated alongside microglia in brain injury [55][56]. Moreover, increasing evidence point to the importance of astrocyte-microglia communication in the pathogenesis of neurodegenerative diseases [28][57]. While we have found that both astrocytes and microglia are activated early in disease, it is unclear whether microglial NF-κB signaling is required or contributes to astrogliosis in SCA1 mice [10].

**Fig 4:**
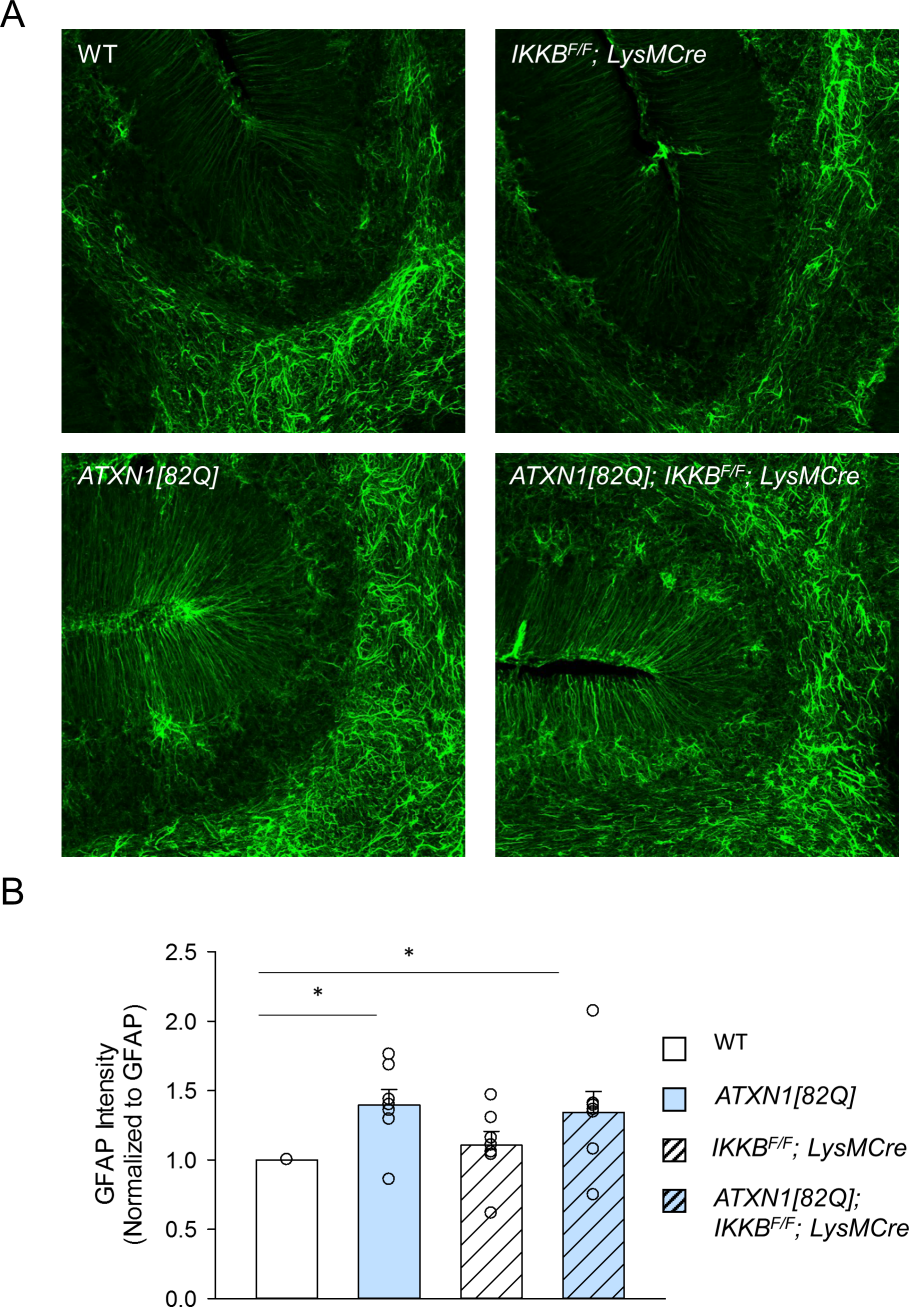
Astroglial GFAP expression in *IKKβ*^*F/F*^*;LysM Cre* and *ATXN1[82Q]; IKKβ*^*F/F*^*;LysM* mice. **A.** Representative image of astrocyte specific GFAP staining **B**. Quantification of GFAP staining in the molecular layer of 3-month old mice, * indicates P < 0.05, Kruskal-Wallis test with Dunn’s test, N = 6 per each genotype). Each dot represents one mouse and open bars are WT controls, light blue bars are *ATXN1[82Q]* mice, and hashed bars indicate the presence of *IKKβ*^*F/F*^*;LysM Cre*.

Therefore, we next examined whether cerebellar astrogliosis is altered in *ATXN1[82Q]; IKKβ*^*F/F*^*;LysM Cre* mice. Astrogliosis was examined using immunohistochemistry of parasagittal cerebellar slices with the antibody against the marker of astrogliosis, glial fibrillary acidic protein (GFAP)(**Fig 4A**) [10]. As we have previously demonstrated there was an increase in the intensity of GFAP staining in the cerebella of *ATXN1[82Q]* mice compared to their wild-type littermates, that was not significantly altered in *ATXN1[82Q];IKKβ*^*F/F*^*;LysM Cre* mice. Moreover, *IKKβ*^*F/F*^*; LysM Cre* mice were not different from control WT mice (**Fig 4B**, normalized to N = 6 WT littermate controls *IKKβ*^*F/F*^*;LysM Cre* mice 1.04 ± 0.17, N = 6, *ATXN1[82Q]* mice 1.33 ± 0.18, N = 6, *ATXN1[82Q]; IKKβ*^*F/F*^*;LysM Cre* mice 1.39 ± 0.27, N = 6). Classically activated microglia have recently been found to induce expression of neurotoxic astroglial A1 phenotype [58]. However, we have found only a trend towards lower expression of A1 astroglial genes, such as decreased expression of complement C3 (*ATXN1[82Q]* mice 1.78 ± 0.28, N = 4, *ATXN1[82Q]; IKKβ*^*F/F*^*;LysM Cre* mice 1.48 ± 0.21, N = 4 normalized to N = 4 WT littermate controls, data not shown, P > 0.05 one-way Kruskal-Wallis test with Dunn’s test).

### Purkinje neuron somatic synapse puncta are retained in *IKKβ*^*F/F*^*;LysM Cre* mice

While *IKKβ*^*F/F*^*;LysM Cre* mice demonstrate profound motor deficits on rotarod, we did not detect any overt degeneration in Purkinje neurons or activation of cerebellar astroglia (**Fig 2, S3 Fig**). Since pharmacological depletion of microglia in adult mice, did not cause any motor deficits [50], we reasoned that motor deficits seen in *IKKβ*^*F/F*^*;LysM Cre* mice may be due to a developmental effect. To establish the appropriate synaptic connections during brain development it is critical to balance synaptogenesis, synapse pruning and maturation. Microglia are critical during this developmental time period where they serve to remove apoptotic cells and unnecessary synapses in order to establish functional neural circuits [17][59]. In cerebellum, shortly after birth, there are multiple climbing fiber (CF) synapses on the soma of Purkinje neurons that are removed during postnatal cerebellar development from P7-P21 [60][61]. Thus, in adult cerebellum approximately only one CF contacts proximal dendrites but not the soma of Purkinje neurons [62][63]. Furthermore, previous studies have shown that reduced synaptic pruning during development and the persistence of somatic puncta on Purkinje neurons impairs motor coordination in mice [64][62].

**Fig 5:**
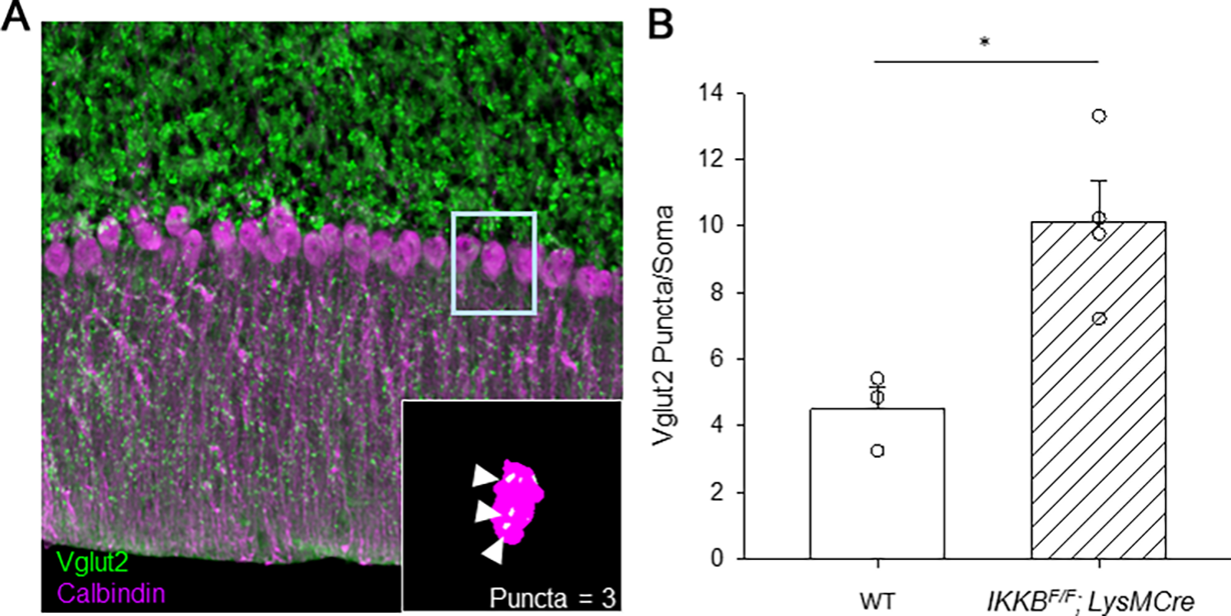
Quantification of the climbing fiber puncta on the soma of Purkinje neurons in *IKKβ*^*F/F*^*;LysM Cre* mice. **A.** Cerebellar slices were co-stained with Calbindin (purple) and VGLUT2 (green). **Inset** An example of puncta counting. **B.** Average number of somatic puncta on Purkinje neurons. * P < 0.05, Student’s t-test.

Since *LysM* promoter is active from E7 [45] it has potential to alter NF-κB signaling and thereby microglial function in synaptic pruning during development. We therefore tested whether there is a retention of somatic puncta in *IKKβ*^*F/F*^*;LysM Cre* mice that could underline the found motor dysfunction. To quantify Purkinje neuron’s somatic synapse puncta, we co-stained cerebellar slices with calbindin and a marker of climbing fiber terminals vesicular glutamate transporter 2 (VGLUT2). By using in house written ImageJ macro to objectively quantify the number of somatic puncta, we found that *IKKβ*^*F/F*^*;LysM Cre* mice had significantly more unpruned CF terminal puncta on the soma of Purkinje neurons than WT mice (**Fig 5,** WT 4.5 ± 0.6, N = 3, *IKKβ*^*F/F*^*;LysM Cre* mice 10.12 ± 0.24, N = 4, student’s t-test, P = 0.0156). We have found similar results in *IKKβ*^*F/WT*^*;LysM Cre* mice (**S5 Fig**). This result may suggest that the canonical NF-κB pathway in microglia has important role in pruning somatic synapses on Purkinje neurons during cerebellar development.

### LysM Cre is not selective for microglia

**Fig 6:**
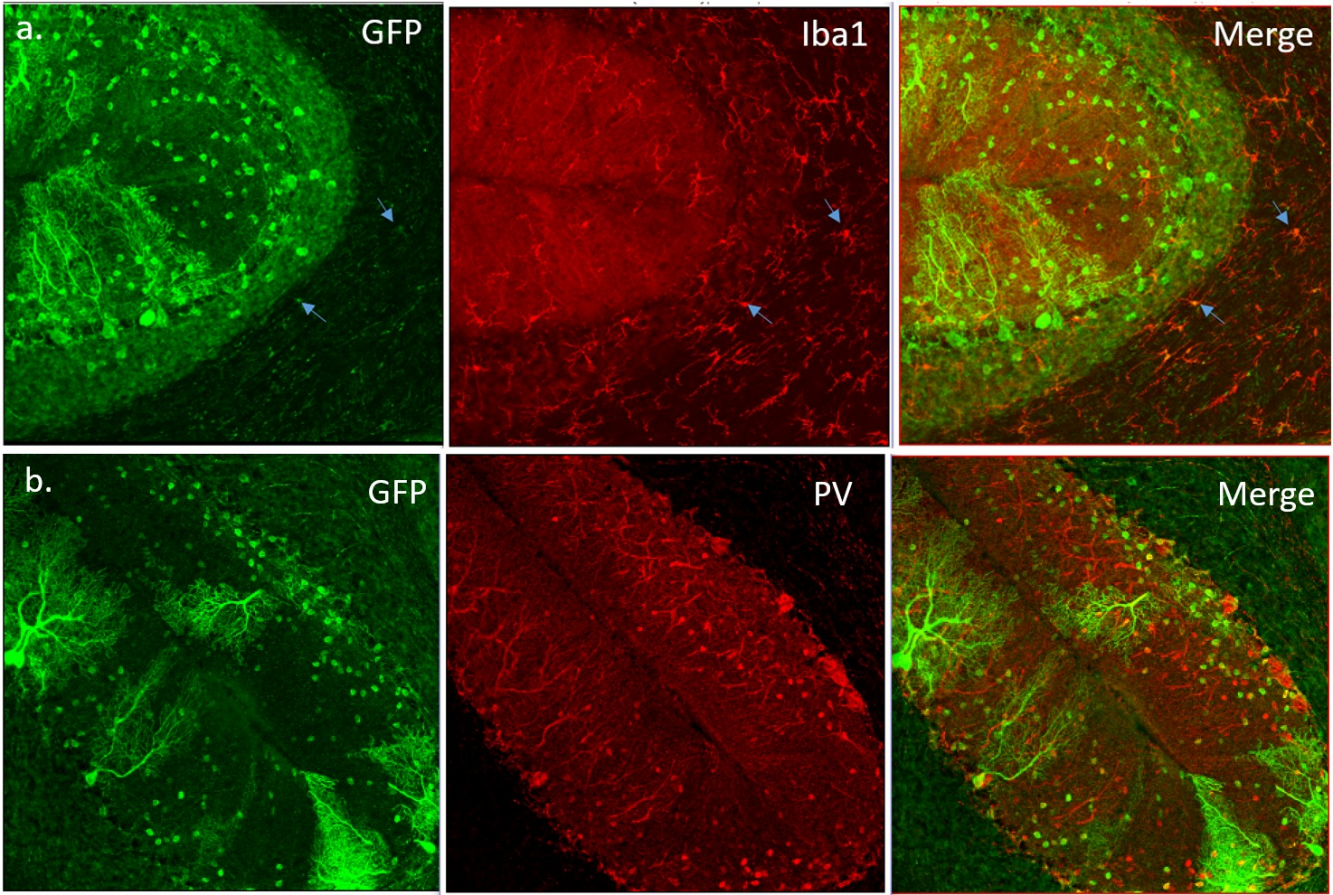
LysM Cre activity is not restricted to microglia cells. Cerebellar slices from *LysM-Cre GCamp6* mice were co-stained for GFP, indicating Cre activity and markers of microglia (Iba1) and inhibitory interneurons [Parvalbumin (PV)]. **A.** Representative confocal images of GFP and Iba1 co-staining. Arrows indicate cells that are both GFP positive and Iba1 positive. Around 10% Iba1 positive cells are also GFP positive. **B.** Representative confocal images of GFP and PV co-staining. Around 20% PV-positive cells are also GFP positive.

To test the specificity and efficiency of Cre activation in microglia, we crossed *LysM Cre* mice with the reporter *GCaMP6* mice [65]. These reporter mice have a floxed-STOP cassette preventing transcription of the GCaMP6 (that consists a green fluorescent protein GFP fused to a calcium binding calmodulin and M13 protein domains). Cre recombinase in LysM cells should excise STOP cassette thereby leading to GCaMP6 expression and GFP immunoreactivity. Cerebellar slices from *GCaMP6;LysM Cre* mice were stained against GFP (green) and microglial maker Iba1 (**Fig 6A)**. Only around 10–15% of cerebellar microglia were co-labeled with GFP and Iba1. This low microglial efficiency of LysM Cre line was previously demonstrated [44] and may contribute to the lack of changes in SCA1 severity in *ATXN1[82Q];IKKβ*^*F/F*^*;LysM Cre* mice. Moreover, we were surprised to detect GFP positive cells in the molecular and Purkinje cell layers that were co-labeled with parvalbumin (PV), a marker of GABA-ergic interneurons. We found that approximately 20% of GFP positive cells in the molecular layer are also PV-positive interneurons and Purkinje cells (**Fig 6B)**. We tried to confirm these results with IKKβ immunohistochemistry of cerebellar slices, but we could not achieve good staining with several commercially available anti-IKKβ antibodies. Thus, it is possible that reduced pruning and motor deficits in *IKKβ*^*F/F*^*; LysM Cre* mice may be caused by loss of IKKβ in cerebellar neurons.

## Discussion

We have found that *ATXN1[82Q];IKKβ*^*F/F*^*;LysM Cre* mice have reduced microglial density and TNFα expression in cerebellum, but undistinguishable motor and cerebellar neurological phenotype compared to *ATXN1[82Q]* mice. Moreover, we have found motor deficits in *IKKβ*^*F/F*^*;LysM Cre* mice that were dissociated from overt Purkinje cell degeneration but instead, associated with abnormal retention of climbing fiber synapses on Purkinje soma. These results may indicate that microglial NF-κB signaling is important for pruning of immature surplus synapses during cerebellar development, but it is not critical to the pathogenesis of SCA1.

Microglia are the resident phagocytic cells of the brain that derive from primitive myeloid progenitors in yolk sac and populate the brain during early embryonic development. As such, microglia are well positioned to influence brain development and sculpt functional neuronal connections, including elimination of synapses and neurons. Pruning of immature surplus synapses is critical for the establishment of the appropriate synaptic connections during brain development [17][59]. In cerebellum, shortly after birth, there are multiple climbing fibers (CFs) that contact Purkinje neuron soma, while in adult cerebellum one climbing fiber contacts proximal dendrites but not soma of Purkinje neurons [55]. This change in wiring is achieved during postnatal days (P) 7–21 when one “winning” CF translocate synapses to the proximal dendrites of Purkinje neurons while CF synapses on the soma of Purkinje neurons are pruned [63]. Since previous studies have shown that reduced synaptic pruning during development and ensuing persistence of somatic puncta on Purkinje neurons can impair motor coordination in mice [64][62], it is possible that observed persisting somatic CF synapses in Purkinje neurons in adult *IKKβ*^*F/F*^*;LysM Cre* mice could contribute to motor deficits exhibited by these mice. While role of microglia in the cerebellar development is not well understood [18], our novel results may suggest that the canonical NF-κB pathway in microglia, typically considered as pro-inflammatory, is required for the removal of immature synapses during cerebellar development.

In chronic disease microglia are thought to be neurotoxic in part due to the increased production of pro-inflammatory cytokine TNFα. TNFα is one of the key microglial cytokines that modulates synaptic strength in response to neuronal activity in physiological conditions [66]. As different levels of TNFα are known to increase or decrease synaptic strength, regulation of its expression in microglia is tightly regulated, in part through NF-κB signaling [67][68]. In our previous experiments we have demonstrated that microglial density is increased pre-symptomatically in SCA1 mice concurrent with increased production of TNFα [10]. Moreover, we have found that pharmacologically depleting microglia early in SCA1 reduced production of TNFα [50], indicating that microglia produce most of TNFα in SCA1 mice. However, despite the observed decrease in microglial density and expression of TNFα, SCA1 motor and neurological pathology were not ameliorated in *ATXN1[82Q];IKKβ*^*F/F*^*;LysM Cre* mice. While these results may suggest that microglia and TNFα expression are not critical for the pathogenesis of SCA1 during early stages of disease, it is important to note that neither microglial density nor TNFα expression were fully reversed.

One likely reason for the partial reduction of neuroinflammation and the lack of differences between *ATXN1[82Q]* and *ATXN1[82Q];IKKβ*^*F/F*^*;LysM Cre* mice is the low efficiency of *LysM Cre* line. Using reporter mice we have found that approximately 15% of cerebellar microglia showed Cre activity as was previously demonstrated [44]. Thereby, it is possible that inhibition of microglial NF-κB pathway is beneficial in SCA1, but that due to the low efficiency of Cre recombination in *LysM Cre* line, we did not reach the threshold of NF-κB inhibition necessary for the significant alteration of disease phenotype.

However, it is important to note that this low efficiency of *LysM Cre* line was sufficient to modulate pathogenesis is mouse models of several conditions, including AD [69][48], Rett syndrome [46], multiple sclerosis [70], ischemia [44], obesity [71], and depression [72]. For example, genetic deletion of IKKβ in microglia ameliorated neuronal loss in a mouse model of excitotoxicity and ischemic brain injury [44]. LysM Cre deletion of IKKβ in the microglia of mouse model of multiple sclerosis, experimental autoimmune encephalomyelitis (EAE) mice, delated the onset and alleviated the severity of EAE neurological symptoms, such as ataxia and paralysis of limbs [73]. Furthermore, *LysM Cre* mice were used to demonstrate that microglial progranulin deficiency increases plaque deposition and impairs phagocytosis in AD [74] and that targeted expression of MECP2 in myeloid cells, driven by *LysM Cre* promoter in an Mecp2-null background attenuated pathology in a mouse model of Rett syndrome [46]. Thus, because low efficiency of *LysM Cre* had a significant effect in these other diseases, it is also possible the role of microglia in pathogenesis may be specific to each disease or to the stage of disease [75]. For example Liao et al. demonstrated that in mouse model of ALS microglia have neuroprotective role at disease onset, whereas at the end-stage of disease microglia have a neurotoxic role, supporting the transformation of microglial phenotype during disease progression [75].

In this study we have focused on the early stages on SCA1, due to their significant therapeutic potential. It is possible that role of microglial neuroinflammation in the pathogenesis of SCA1 may change as the extent of injury worsens with disease progression. Future studies testing the role of microglial NF-κB signaling in the late stage of SCA1 using inducible Cre-LoxP system are needed to address this question. However, cerebellar microglia are not well studied and it is also possible that cerebellar microglia may play a different role in disease than microglia in other brain regions. Genome-wide analysis of microglia from different brain regions demonstrated that cerebellar microglia exist in a more immune-vigilant state that is further augmented during aging [43]. Time-lapse in vivo imaging, found that cerebellar microglia have decreased parenchymal surveillance compared to cortical microglia [76]. Thus our data may also indicate that cerebellar microglia and in particular NF-κB signaling behave differently in disease compared to microglia from other brain regions.

Another possible explanation for the observed lack of amelioration of disease phenotype in *ATXN1[82Q];IKKβ*^*F/F*^*;LysM Cre* mice is that reactive astrocytes have a predominant role in contributing to the pathogenesis of SCA1. Astrocytes have numerous important functions in the brain, including structural and metabolic support of neurons, and regulation of extracellular ion and neurotransmitter homeostasis [77][78]. Similar to microglia, astrocytes also undergo activation in neurological diseases that results in morphological and functional changes, including enlarged cell bodies, thickening of cell processes, and alteration in their ability to maintain homeostasis and to provide neurotrophic support. Liddelow et al. demonstrated that LPS activated microglia induce neurotoxic phenotype of astroglia though the secretion of pro-inflammatory cytokines, including TNFα [58]. We have not detected a significant alteration of astrogliosis, measured as increased expression of GFAP and A1-associated genes in *ATXN1[82Q];IKKβ*^*F/F*^*;LysM Cre* cerebella, indicating that reduced microglial density and TNFα expression may not be sufficient to affect astrogliosis and its possible neurotoxic effects in SCA1 mice.

Finally, when interpreting disease phenotype in *ATXN1[82Q]; IKKβ*^*F/F*^*;LysM Cre* mice we need to consider specificity of the *LysM Cre* line [45][79][80][74]. We examined the cerebellar cell specificity of the *LysM Cre* line, by crossing them with the reporter line [65], and were surprised to find evidence of Cre recombination not only in microglia as expected, but also in neuronal cells including Purkinje neurons and parvalbumin (PV) positive interneurons. Thus, it is possible that a decrease in microglial NF-κB and neuroinflammation in the cerebellum may have a beneficial effect on SCA1 pathogenesis, but this is masked by a negative effects of decreased NF-κB in Purkinje neurons [81]. Similarly, while the observed retention of climbing fiber puncta on the soma of Purkinje neurons is sufficient to cause motor deficits in *IKKβ*^*F/F*^*;LysM Cre* mice, it is possible that decreased NF-κB activity in Purkinje neurons contributes to the these motor deficits.

In addition, while efficiency of LysM Cre mediated deletion may be higher in the cerebellum compared to the other regions of the brain [82], it is by no means limited to the cerebellum. Thus, it is possible that loss of IKKβ in other brain regions contributes to the impaired motor phenotype of *IKKβ; LysM Cre* mice. We have not found any change in microglial density or morphology in the striatum, a brain region involved in the control of movement, but we cannot exclude subtle changes in microglial function. To resolve these issues of cell and region specificity of IKKβ depletion future studies using more selective Cre lines or AAV viruses expressing Cre recombinase to selectively target cerebellar microglia, Purkinje neurons or PV interneurons are needed. Moreover, previous studies, including the comprehensive evaluation of several myeloid-expressing Cre strains, have demonstrated LysM-Cre mediated deletion in macrophages, neutrophils, and monocytes [83][84]. Thus, while depletion of IKKβ in microglia may be beneficial in the context of SCA1 (e.g. by lowering neuroinflammation), it is possible that loss of IKKβ in these other myeloid cells may negatively impact on the motor function in mice and thereby contribute to the unaltered disease severity in *ATXN1[82Q];IKKβ*^*F/F*^*;LysM Cre* mice or to the motor deficits in *IKKβ*^*F/F*^*;LysM Cre* mice. Importantly our study strongly cautions against using *LysM Cre* mice to study microglia specific effects and calls for considering neuronal and myeloid cell recombination when re-interpreting previous CNS studies using these mice.

## Materials and methods

### Mouse lines

*ATXN1[82Q]*, *IKKβ*^*F/F*^ and *LysM Cre* mice were generated as previously described [45][71][47]. Because our previous studies have detected no sex-specific effects in SCA1, we have used an equal mix of animals of both sexes for our experiments. All animal experiments were performed in compliance with the National Institutes of Health’s Guide for the Care and Use of Laboratory Animals and the University of Minnesota Institutional Animal Care and Use Committee.

The protocol was approved by the University of Minnesota Institutional Animal Care and Use Committee (protocol number 1511-33160A).

### Rotating rod test

Motor deficits were assessed using rotarod assay at the age of 3 months. The rotarod test was performed as previously described [85]. Briefly, mice were placed on the rotarod apparatus (Ugo Basile) that accelerates from a speed of 4 rotations per minute (rpm) to 40 rpm over a 5-minute period. We recorded the time it takes for a mouse to fall off rotarod, to a maximum of 10 minutes. Mice were subjected to four trials per day for four consecutive days, with at least ten minutes of rest between each trial. Data for the performance on day 4 was analyzed using one-way ANOVA with Bonferroni post-hoc test. Significance was assumed at P < 0.05. All tests were performed blinded with respect to the genotype.

### Immunohistochemistry

Mouse brains were fixed overnight in 4% formaldehyde, incubated in 30% sucrose, and cut into 45 μm sections on cryostat (Leica, CM 1850). Sections were washed three times in cold Phosphate Buffered Saline (PBS), and incubated in blocking buffer (3% Normal Donkey Serum in PBST (1% Triton X-100 PBS) for 1 hr. at room temperature (RT). This was followed by an overnight incubation at 4°C in blocking buffer containing the relevant primary antibody [anti-GFAP (Z0334, DAKO), anti-Iba1 (019–19741, WAKO), anti-Calbindin-D-28K (C9848, Sigma Aldrich), anti-VGLUT2 (MAB5504, Millipore), anti-ataxin1 (11NQ, a gift from Dr. Harry Orr)]. After incubation with primary antibody we washed samples three times with PBS, and incubated them overnight at 4°C with the relevant fluorescently labeled secondary antibody (Alexa, Invitrogen). We mounted stained sections on slides with Vectashield mounting media containing DAPI (Vector Laboratories) for imaging at 20X magnification under the confocal microscope (Olympus FV1000). For each mouse, we imaged at least six different, randomly selected cerebellar lobules. Each image consisted of 20 μm Z-stacks, composed of 1μm thick image slices. Images were analyzed using FIJI (ImageJ, NIH) software. For analysis of calbindin length and intensity, we drew a straight line extending from the middle of Purkinje cell body to the end of the molecular layer in at least two places for each lobule. We then used the Measure function in ImageJ to quantify the length of the line to obtain measurement of the width of the molecular layer, and the average intensity along the line to obtain calbindin intensity. For GFAP staining, we used the Measure function in ImageJ to quantify the intensity of signal in the molecular layer that was then normalized to the average intensity in the control group (wild-type mice). For microglial Iba1 staining, we counted the Iba1 positive cells and to obtain microglial density we divided the number of Iba1 positive cells by the area in which they were counted. Staining, microscopy, and image analysis were performed blinded to the experimental groups. Data was analyzed using ANOVA with Bonferroni post-hoc test using GraphPad Prism software (GraphPad software).

For counting puncta, tissue was stained in the same manner using antibodies against calbindin and VGLUT2 and then imaged at 60X magnification (20 μm Z stack, 1 μm Z step). Images were then processed using an in-house written ImageJ macro that would segment the calbindin and VGLUT2 signals, and procedurally count VGLUT2 puncta (minimum size 1.4 μm^3^, maximum μm^3^ 70.3 size filter for puncta) on somata of Purkinje cells, defined by volume segmentation (minimum/maximum volume 281.3 and 7,031.3 μm^3^ respectively). Resultant images of segmented somata and counts were filtered by hand to remove artefactual somata. Counts were compared using a student’s t test.

### Quantitative real time RT-PCR

Total RNA was isolated from cerebella using TRIzol (Invitrogen) and treated with DNAse (TURBO Dnase, Thermo Fisher Scientific). Complementary DNA (cDNA) synthesis was performed in duplicate, using Superscript III First-Strand Synthesis SuperMix (Invitrogen) and random hexamer primers. The expression level of each gene was determined on a Light Cycler 480 II (Roche) using Light Cycler 480 SYBR Green PCR I Master mix (Roche). Cycling conditions were: 5 min at 95°C, followed by 40 cycles of 95°C for 15 sec, 56°C for 1 min. Samples were analyzed in triplicate and a melting curve analysis was performed in each sample at the end of the qPCR reaction to confirm specificity of reaction. Expression levels of mouse 18S RNA (forward primer: 5’AGT CCC TGC CCT TTG TAC ACA 3’ and reverse primer: 5’ CGA TCC GAG GGC CTC ACT A -3’) were used as internal controls. Primers used for the Purkinje neuron’s Magenta cluster genes (*calbindin*, *ITPR*, *INPP5a*, *Garnl3*, *Pcp4*, *Homer3* and *Rgs8*) were from Ingram et al. [51], and primers used for astroglial genes (*Kir4*.*1*, *EAAT1*, *P2RY*, *Aqp4*, *C3*, *S100*, and *Glul)* and microglial gene (*TNFα*) were PrimeTime qPCR primers (IDT). Relative gene expression was determined by the 2^-ΔΔCt^ method [86]. The threshold cycle (Ct) value was determined for target genes and the endogenous internal controls (18S RNA) in each sample. The difference between target gene and 18S RNA control Ct values was determined for each sample, resulting in the ΔCt value. The ΔCt of a calibrator, a wild type sample, was subtracted from each sample’s ΔCt to yield the ΔΔCt value. Relative fold change was calculated as 2^-ΔΔCt^. Data was analyzed using ANOVA with Bonferroni post-hoc test using GraphPad Prism software (GraphPad software).

### Western blotting

Cerebella were dissected from mice and lysed in RIPA lysis buffer [50mM Tris HCl, pH7.4, 150mM NaCl, 1% sodium deoxycholate, 1% NP-40, 0.2% SDS, phosphatase (Sigma) and protease inhibitors cocktail (Roche)]. After three cycles of freeze and thaw, proteins were separated on a 12% or 15% SDS-PAGE gel and transferred onto a nitrocellulose membrane. The following primary antibodies were used: anti-ATXN1 (rabbit 11NQ, Orr lab), anti-PSD95 (Biolegend), and alpha-tubulin (mouse, Sigma). Signals from secondary antibodies linked to horseradish peroxidase (HRP) (GE Healthcare) were detected using Amersham ECL Western Blotting Detection Reagent (GE Healthcare) and ImageQuant LAS 4000 imager (GE Healthcare); protein levels were quantified using ImageQuant (GE healthcare) and ImageJ. Data was analyzed with one-way ANOVA followed by Bonferroni post-hoc test.

### Statistical analysis

Statistical tests were performed with GraphPad Prism or R. For rotarod, we have used one-way ANOVA followed by either Tukey’s HSD or Bonferroni post-hoc test. For IHC, qRT-PCR, and Western blot quantification, we have also used Kruskal-Wallis test with Dunn’s test or one-way ANOVA followed by Bonferroni post-hoc test.

## Supporting information

S1 Fig A novel SCA1 transgenic mouse line *ATXN1[82Q]; IKKβ*^*F/F*^*;LysM Cre* was created to express Cre-recombinase specifically in microglia under the control of *LysM* promoter, and to remove loxP-enclosed exon 3 of IKKβ from microglia. (TIF)

S2 Fig Microglial density in the striatum of *IKKβ*^*F/F*^*;LysM Cre* mice. **A.** Brain slices from *IKKβ*^*F/F*^*;LysM Cre* and control WT littermates were stained with Iba1 at three months of age. Insets show magnified images of microglia. **B.** Quantification of microglial density in the molecular layer (N ≥ 3 per each genotype), Student’s t-test P = 0.9008. Each dot represents one mouse, and values indicate mean ± SEM.
(TIF)

S3 Fig Motor function in *IKKβ*^*F/WT*^*;LysM Cre* mice. *IKKβ*^*F/WT*^*;LysM Cre* and control WT littermates were tested on a rotarod at three months of age. Each dot represents one mouse, and values indicate mean ± SEM, * indicates P < 0.05 by one-way ANOVA with Bonferroni post-hoc test.
(TIF)

S4 Fig Ataxin-1 and PSD 95 protein levels in *ATXN1[82Q]; IKKβ*^*F/F*^*;LysM* mice. Ataxin-1 (**A**) and PSD95 (**B**) protein levels were examined using western blotting of cerebellar lysates from 3-month-old mice. Each dot represents one mouse, and values indicate mean ± SEM, data was analyzed using one-way ANOVA followed by Bonferroni post-hoc test.
(TIF)

S5 Fig Reduced synaptic pruning in the cerebella of *IKKβ*^*F/WT*^*; LysM Cre* mice. **A**. Cerebellar tissue stained with Calbindin (red) and VGLUT2 (green). **B.** Average number of somatic puncta on Purkinje neurons. * Student’s t-test P < 0.05.
(TIF)

## Abbreviations

ATXN1: ataxin1
AD: Alzheimer disease
ALS: amyotrophic lateral sclerosis
GFAP: glial fibrilarly acidic protein
HD: Huntington disease
Iba1: ionized calcium-binding adapter molecule 1
PD: Parkinson disease
PN: Purkinje neuron
SCA1: Spinocerebellar ataxia type1
TNFα: Tumor necrosis factor alpha
