# Supplementary figures and images for: Inhibition of NF-κB signaling in IKKβF/F;LysM Cre mice causes motor deficits but does not alter pathogenesis of Spinocerebellar ataxia type 1
Source: PLoS One. 2018 Jul 5;13(7):e0200013. doi: 10.1371/journal.pone.0200013 (PMC6033432; doi:10.1371/journal.pone.0200013)

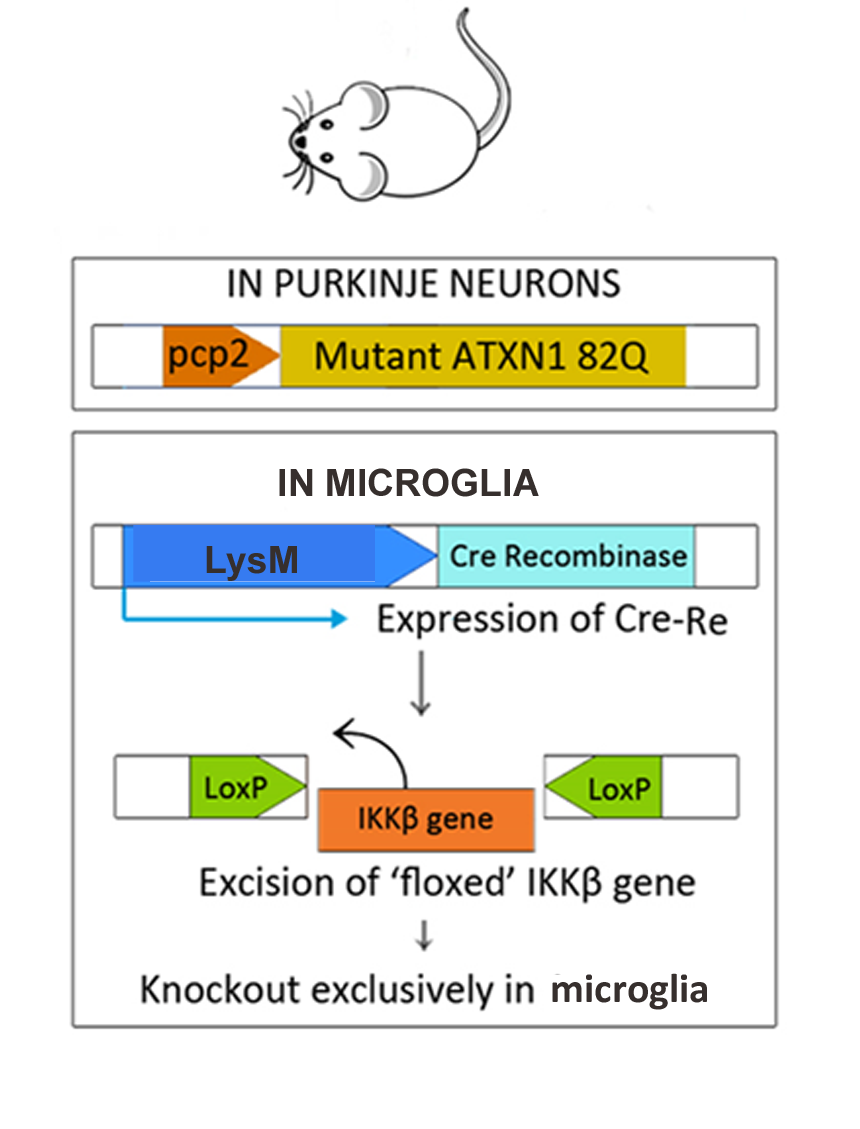

Supplement: S1 Fig — (TIF) [file pone.0200013.s001.tif]

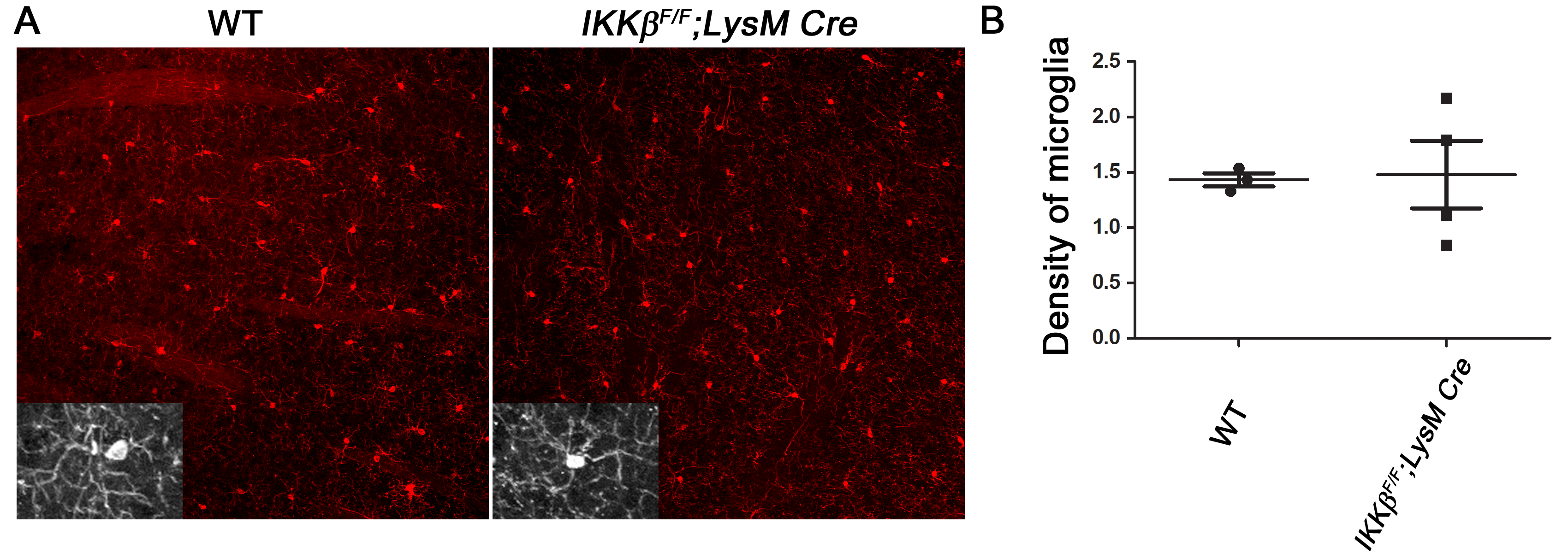

Supplement: S2 Fig — A. Brain slices from IKKβF/F;LysM Cre and control WT littermates were stained with Iba1 at three months of age. Insets show magnified images of microglia. B. Quantification of microglial density in the molecular layer (N ≥ 3 per each genotype), Student’s t-test P = 0.9008. Each dot represents one mouse, and values indicate mean ± SEM. (TIF) [file pone.0200013.s002.tif]

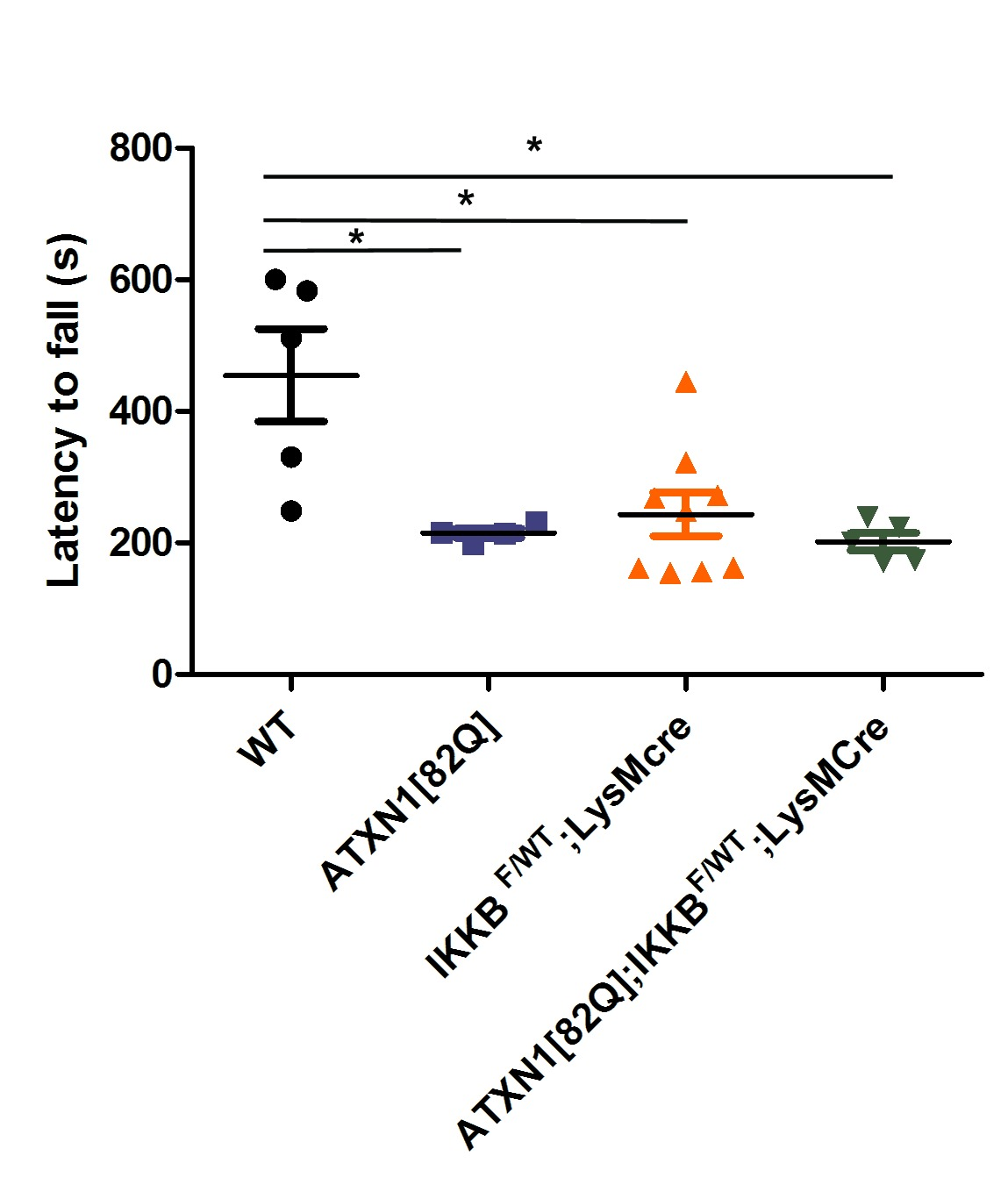

Supplement: S3 Fig — IKKβF/WT;LysM Cre and control WT littermates were tested on a rotarod at three months of age. Each dot represents one mouse, and values indicate mean ± SEM, * indicates P < 0.05 by one-way ANOVA with Bonferroni post-hoc test. (TIF) [file pone.0200013.s003.tif]

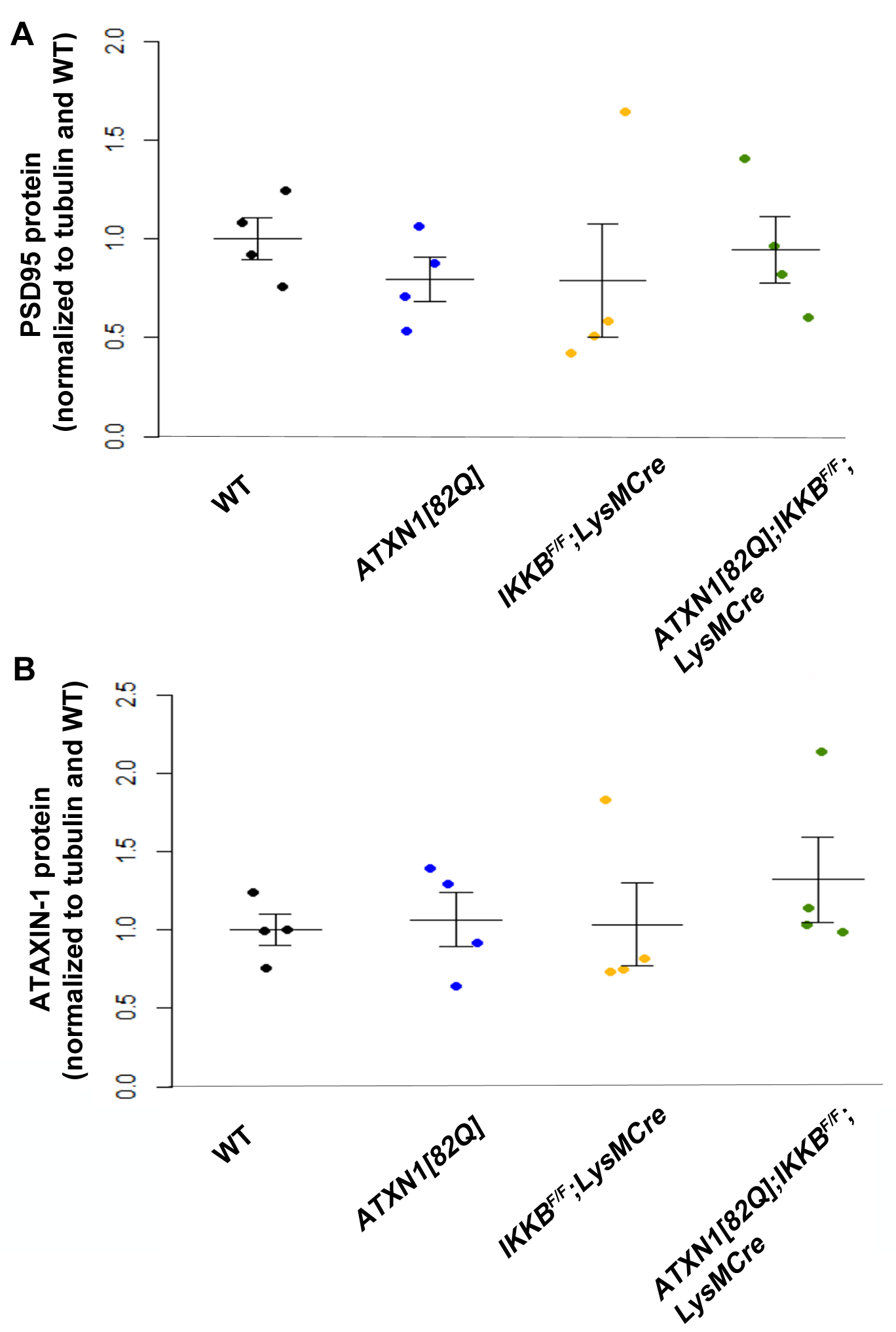

Supplement: S4 Fig — Ataxin-1 (A) and PSD95 (B) protein levels were examined using western blotting of cerebellar lysates from 3-month-old mice. Each dot represents one mouse, and values indicate mean ± SEM, data was analyzed using one-way ANOVA followed by Bonferroni post-hoc test. (TIF) [file pone.0200013.s004.tif]

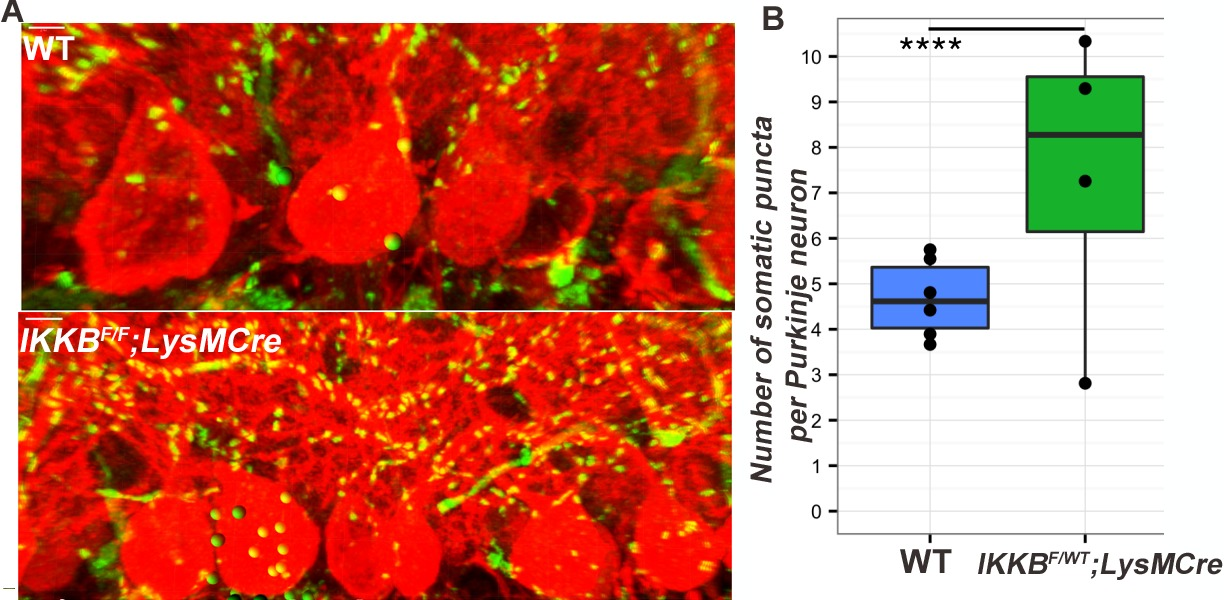

Supplement: S5 Fig — A. Cerebellar tissue stained with Calbindin (red) and VGLUT2 (green). B. Average number of somatic puncta on Purkinje neurons. * Student’s t-test P < 0.05. (TIF) [file pone.0200013.s005.tif]
